# Evaluation of immune cell markers in tumor tissue treated with radioimmunotherapy in an immunocompetent rat colon carcinoma model

**DOI:** 10.1186/s13550-015-0126-y

**Published:** 2015-09-15

**Authors:** Erika Elgström, Sophie E. Eriksson, Otto Ljungberg, Pär-Ola Bendahl, Tomas G. Ohlsson, Rune Nilsson, Jan Tennvall

**Affiliations:** Department of Clinical Sciences Lund, Division of Oncology and Pathology, Lund University, Barngatan 2B, SE-221 85 Lund, Sweden; Department of Laboratory Medicine, Clinical Pathology, Skane University Hospital, Lund, Sweden; Department of Medical Radiation Physics, Lund University, Lund, Sweden; Department of Oncology, Skane University Hospital, Lund, Sweden

**Keywords:** Radioimmunotherapy, CD2, CD3, CD8, CD68, CD163, Immune cells, Immunocompetent, Tumor microenvironment, Immune rejection

## Abstract

**Background:**

Immune cells within the tumor can act either to promote growth or rejection of tumor cells. The aim of the present study was to evaluate immune cell markers (number and localization) within the tumor before and during rejection due to radioimmunotherapy, to determine whether there is a change in markers related to rejection and/or tolerance of the tumor cells.

**Methods:**

Thirty immunocompetent rats were inoculated with syngeneic rat colon carcinoma cells and 13–14 days later 21 of these rats were treated with 400 MBq/kg of ^177^Lu-DOTA-BR96 monoclonal antibodies. The treated animals were sacrificed and dissected 1, 2, 3, 4, 6, and 8 days post-injection in groups of three animals per day (6 animals on day 8); while the nine untreated animals were sacrificed and dissected on day 0. Paraffin sections were used for immunohistochemical staining of CD2, CD3, CD8α, CD68, and CD163 antigens. Positive cells were counted within: vital tumor cell areas, necrotic areas, granulation tissue surrounding and between the tumor cell areas. The change in the number of positive cells over time in tumors treated with radioimmunotherapy in the same location was evaluated with linear regression models. The number of positive cells in various locations and the number of various antigen-positive cells within the same location were also evaluated over time using box plots.

**Results:**

There were a higher number of cells expressing immune cell markers in granulation tissue compared with vital tumor cell areas. Cells expressing markers decreased during radioimmunotherapy, and T-cell markers decreased more than macrophage markers in tumors treated with radioimmunotherapy. The expression of CD8α was higher than that of the other T-cell markers evaluated (CD3 and CD2), which could be explained by the additional expression of CD8α by natural killer (NK) cells and a subset of dendritic cells (DCs). The expression of CD68 (all macrophages, DCs, and neutrophils) tended to be higher than that of CD163 (pro-tumor macrophages).

**Conclusions:**

In this model, we demonstrated a higher number of positive cells for immune cell markers related to augmenting the immune rejection than immune tolerance of tumor cells in tumors and a decrease in markers during radioimmunotherapy.

## Background

Distant metastases are the main reason that patients die from cancer [1]. It is thus important to identify patients with a high risk of developing metastases and try to prevent the development of tumor cells into metastases, since metastases can develop after years of undetectable disease. This has been explained by escape from dormancy using, for example, immunosurveillance [2–6]. This means that tumor cells are present but do not evolve into a detectable tumor due to the action of the immune system. If the immune response fails to suppress the growth of tumor cells, they will evolve into a detectable tumor. It would be informative to evaluate the infiltration of immune cells in the primary tumor [7–11], since it has been shown that immunoscore classification of tumors is a clinically useful prognostic factor [12, 13]. Immunotherapeutic strategies aiming to overcome the immune tolerance of tumor cells are a new promising approach. An example of this is the use of an immune checkpoint inhibitor (e.g., ipilimumab) directed against the cytotoxic T lymphocyte antigen (CTLA-4), which has been found to improve the survival of patients with metastatic melanoma [14–16].

Our rat colon carcinoma model is syngeneic, as the cell line used was established in the same rat strain as used in the experiments. The animals are fully immunocompetent making the model more clinically relevant. In a previous study on this immunocompetent syngeneic rat colon carcinoma model, we demonstrated that treatment with ^177^Lu-labeled monoclonal antibodies (radioimmunotherapy, RIT) resulted in local complete response (CR) in the majority of the animals (17 of 19) within 2 weeks [17]. However, half of the animals developed distant metastases during the follow-up period of 100 days post-injection (p.i.).

The therapeutic effect of RIT is derived from both the antibody and the decay of the radionuclide (in this case ^177^Lu). To the best of our knowledge, no studies have been carried out to evaluate the effects of RIT on the infiltration of immune cells into tumors. The aim of the present study was, thus, to evaluate immune cell markers (both number and localization) within the local tumor at the time of treatment and during rejection due to RIT, in order to determine whether there is any change in the markers related to rejection and/or tolerance of the tumor cells.

## Methods

### The syngeneic animal model

BN7005-H1D2 is a cell line established from a 1,2-dimethylhydrazine-induced colon carcinoma in a Brown Norway (BN) rat [18]. We determined the radiosensitivity of this cell line, expressed as the fraction of survival after 2 Gy to be 0.5 (^137^Cs radiation source, unpublished data). This is similar to the radiosensitivity of human colorectal carcinoma cell lines [19]. The cells were cultured in RPMI-1640 medium supplemented with 10 % fetal calf serum (both from PAA Laboratories GmbH, Pasching, Austria), 1 mM sodium pyruvate, 10 mM HEPES buffer, and 14 mg/L gentamicin (all from Gibco, Invitrogen, Carlsbad, CA) at 37 °C, in a humidified environment containing 5 % CO_2_. The cells were washed in PBS and detached by treatment with trypsin (both from PAA Laboratories GmbH).

BN rats are immunocompetent and express the target antigen (Lewis Y, Le^y^) in normal tissues, mainly in the epithelium of the gastrointestinal tract [20], similar to humans [21]. The animals were inoculated with 3 × 10^5^ cells between the peritoneum and the abdominal wall under general anesthesia (isoflurane, Abbott Scandinavia AB, Solna, Sweden). Tumor volumes were calculated as tumor length × tumor width^2^ × 0.4 [22]. All experiments were conducted in compliance with European legislation on animal welfare and were approved by the Regional Animal Ethics Committee (Malmö/Lunds djurförsöksetiska nämnd). The animals were housed under standard conditions and fed with standard pellets and fresh water ad libitum.

### The radioimmunoconjugate

The chimeric (mouse/human) monoclonal IgG1 antibody BR96 (Seattle Genetics Inc., Seattle, WA) binding to the Le^y^ was employed. The tumor-associated antigen Le^y^ is expressed on the majority of human epithelial tumors. The dissociation constant between BR96 and the cell line used is 4 nM [23], illustrating its strong binding affinity.

Conjugation of BR96 and DOTA was performed according to Forrer et al. [24]. Briefly, BR96 was transferred to 0.2 M sodium carbonate buffer, pH 9.5, by repeated centrifugation using an Amicon Ultra-15 filter (MW 30 000, Millipore, Billerica, MA). All empty vials were pretreated with 1 % HNO_3_ and all buffers were pretreated with Chelex-100 (BioRad, Hercules, CA) to remove metals. The DOTA chelate (S-2-(4-isothiocyanatobenzyl)-1, 4, 7, 10-tetraazacyclododecane tetraacetic acid; Macrocyclics, Dallas, TX, 2 mg/mL H_2_O) was added to the BR96 antibody (100 mg/mL) at a molar ratio of 3:1 (DOTA:BR96) and incubated for 1 h at 37 °C. The conjugate was purified by repeated centrifugation using an Amicon Ultra-15 filter and transferred to 0.25 M ammonium acetate buffer, pH 5.3, and the final concentration was adjusted to 10 mg/mL BR96.

MALDI-MS was used to determine the number of DOTA moieties per BR96 molecule, by desalting the sample to 18 MΩ · cm H_2_O using a centrifugation filter device, and dividing the increase in molecular mass by 688 (the molecular mass of the DOTA chelate).

The immunoreactivity (*i.e.* the antigen-binding properties) of DOTA-BR96 relative to BR96 was determined from a saturation binding curve, using BN7005 cells as the target antigen. Briefly, increasing concentrations of BR96 and DOTA-BR96 (40 μg/mL–40 mg/mL) were added to the cell plate in triplicate and incubated for at least 90 min. The bound BR96/DOTA-BR96 conjugates were detected with rabbit anti-human IgG-HRP (Dako, Glostrup, Denmark), and the equilibrium binding constant (K_d_) was calculated using Prism 5.02 software (GraphPad Software Inc., binding saturation-one site total, non-specific binding and background constrained to a constant value of zero). The immunoreactivity was given by the ratio of the binding constants: *K*_*d*_(BR96)/*K*_*d*_(DOTA-BR96).

Both the DOTA-BR96 conjugate in 0.25 M ammonium acetate buffer and the ^177^LuCl_3_ solution (MDS Nordion, Vancouver, Canada) were preheated to 45 °C for 10 min. The DOTA-BR96 solution was then added to the vial containing the radionuclide and incubated at 45 °C for 15 min. The reaction was quenched with excess DTPA (diethylene triamine pentaacetic acid) for 5 min. The radiolabeled immunoconjugate was diluted in 1 % human serum albumin (HSA, Baxter Medical AB, Kista, Sweden) to prevent radiolysis from affecting the immunoreactivity. The radiochemical purity was determined with instant thin-layer chromatography (ITLC) using a 1 × 9 cm silica-gel-impregnated fiberglass sheet as the solid phase and 0.1 M EDTA as the mobile phase. To analyze the radiochemical purity and to detect signs of aggregation or fragmentation, separation was performed using size-exclusion chromatography and high-performance liquid chromatography (HPLC) (using a 7.8 × 300 mm molecular sieving column, Phenomenex SEC S3000; Phenomenex, Torrance, CA, eluted with 0.05 M sodium phosphate at 1.0 mL/min).

### Radioimmunotherapy with ^177^Lu-DOTA-BR96

Thirty male BN rats (Harlan, Horst, the Netherlands) were included in the study. Their median weight on the day of administration of RIT (day 0) was 238 g. Twenty-one rats were treated with 400 MBq/kg body weight of ^177^Lu-DOTA-BR96 (150 μg DOTA-BR96 in 0.4-mL saline with 1 % HSA) by intravenous injection in the tail vein 13–14 days after cell inoculation. Our previous study showed that 400 MBq/kg body weight resulted in CR in 17 of 19 animals [17], and that the maximum tolerable activity was 600 MBq/kg body weight [25]. The nine remaining animals were left untreated as a control group. The treated animals were sacrificed and dissected 1, 2, 3, 4, 6, and 8 days p.i. in groups of three animals per day (6 animals on day 8), while the untreated animals were sacrificed and dissected on day 0. The tumors were cut in half, fixed in 4 % paraformaldehyde, and embedded in paraffin.

### Immunohistochemistry

Consecutive 4 μm paraffin sections were used to detect CD2, CD3, CD8α, CD68, and CD163. The characteristics of the antibodies and antigens stained are presented in Table 1. Note that all macrophages express CD68, while CD163 is only expressed by M2 (pro-tumor macrophages) [26, 27].

**Table 1:** Antibody characteristics and antigen expression

Antigen	Clone (supplier)	Dilution	Antigen expression on tumor-infiltrating cells
CD2	OX-35 (AbD Serotec)	1:800	T-cells, B-cells, NK cells
CD3	Polyclonal (18–0102, Invitrogen)	1:200	T-cells
CD8α	OX-8 (AbD Serotec)	1:200	Cytotoxic T-cell, NK cells, DC subset
CD68	ED-1 (AbD Serotec)	1:200	All macrophages, neutrophils, DC, myeloid progenitors (*e.g.* myeloid-derived suppressor cell)
CD163	ED-2 (LSBio, LifeSpan BioSciences)	1:200	Pro-tumor macrophages (M2) [26, 27]

Sections were rehydrated and antigen retrieval was performed using the PT Link pre-treatment (Dako) with Target Retrieval Solution, pH 6 (Dako), preheated to 65 °C. The sections were then heated to 99 °C for 20 min and allowed to cool at room temperature for at least 1 h. The slides were rinsed with distilled water, and endogenous peroxidase was blocked by Peroxidase-Blocking Solution, endogenous biotin by Biotin Blocking System, and proteins by Protein Block Serum-Free (all from Dako). The sections were incubated for 1 (CD2 and CD163) or 1.5 h (CD3, CD8α, and CD68) at room temperature with primary antibody. After washing with Wash Buffer (Dako), the primary antibodies were detected with LSAB2 System-HRP for use on rat specimens (Dako) according to the manufacturer’s instructions. Finally, Liquid DAB+ Substrate Chromogen System (Dako) was used to visualize the antigens before counterstaining with hematoxylin and mounting with Pertex (Histolab, Goteborg, Sweden).

### Evaluation of immunohistochemistry sections

All sections were evaluated blindly by an experienced clinical pathologist (OL). Positive cells were counted in: vital tumor cell areas, necrotic areas, granulation tissue surrounding the tumor cell areas, and between tumor cell areas. The number of positive cells within the high-power field of view of 40× (0.24 mm^2^) was evaluated in two hot spots selected to contain evenly distributed positive cells within the field of view. In views with more than 100 positive cells, the number of positive cells along a diameter of the view was counted. The diameter was chosen randomly. The total number (*n*) of positive cells within the field of view was then calculated using the following equation:

$$ n={\left(\frac{\mathrm{number}\ \mathrm{of}\ \mathrm{positive}\ \mathrm{cells}\ \mathrm{on}\ \mathrm{the}\ \mathrm{diameter}}{2}\right)}^2\times \pi $$

The calculated number of positive cells was confirmed by manual counting in three fields of view, and the calculated value correlated very well with the manually counted values (data not shown).

In some cases, the areas were located in a band-like distribution (mainly granulation tissue) and the areas were not always large enough to cover the whole microscopic field of view. In such instances, the field was oriented in such a way that the area was adjusted to include the diameter of the field of view. The number of positive cells was counted along the diameter and the total number of positive cells was calculated as described above. This procedure was considered necessary to be able to compare density of positive cells over time, between different locations and different immune cell markers.

### Statistics

The change in the number of antigen-positive cells was evaluated using simple linear regression of the log count over time. All immune cell markers (CD2, CD3, CD8α, CD68, and CD163) were individually evaluated in the following locations: vital tumor cell areas, granulation tissue surrounding the tumor cell areas and between tumor cell areas. Multiple linear regression models, including tumor volume on day 0 or tumor volume on the day of sacrifice, were used to evaluate the extent to which the time trends were confounded by tumor volume. Although the linear regression models for the different markers and locations did not always show a good fit to the observed data, they still provided an estimate of trends over time. However, the corresponding regression coefficients should be interpreted as average effects over the follow-up period.

Box plots of ratios of different locations (granulation tissue surrounding the tumor cell areas and between tumor cell areas vs vital tumor cell areas), stratified by time interval, were used to visually compare the number of antigen-positive cells for each antigen. The same technique was use to compare the number of positive cells of different antigens within the same location. Null hypotheses of no difference (ratio 1.0) were evaluated separately for each time interval using the Wilcoxon matched-pairs signed rank sum test.

Animals with no tumor (CR) at the time of sacrifice on days 4 and 6 were not included in the analyses. The scar tissue from tumors on day 8 was collected but could not be included in the calculations since they lacked tumor cell areas.

All statistical calculations were performed using Stata 13.1 (StataCorp LP, College Station, TX).

## Results

### The radioimmunoconjugate

The average number of DOTA molecules conjugated per BR96 antibody was 2.4. The immunoreactivity was 0.9, indicating that the antigen-binding properties did not change as a result of conjugation with DOTA. The radiochemical purity of ^177^Lu-DOTA-BR96 was analyzed using ITLC and found to be 97 %, and less than 1 % of the activity was found in aggregates according to HPLC.

### Tumor sampling

All 30 rats developed local tumors between the peritoneum and the abdominal wall before the day of treatment (day 0). The median tumor volume on day 0 was 1140 mm^3^ (interquartile range 900–1580 mm^3^). The nine untreated control animals were sacrificed, and the tumors were excised on day 0. The animals treated with RIT were sacrificed and the local tumor was excised 1, 2, 3, 4, 6, or 8 days p.i. in groups of 3 rats per day, apart from day 8 p.i. when 6 rats were sacrificed. Tumor tissues could not be sampled in 5 rats due to CR: on day 4 (1 CR), day 6 (1 CR), and day 8 (3 CR) p.i. Scar tissue from tumors was collected from rats showing CR on day 8 p.i.

### T-cell markers—CD2, CD3, and CD8α

**Fig. 1:**
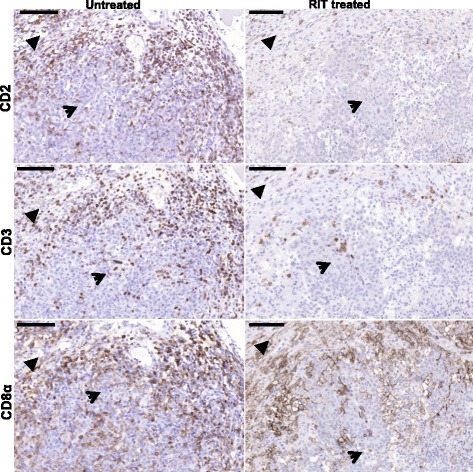
Images of immunohistochemical sections after staining of T-cell markers. CD2 (T-cells, B-cells, NK cells), CD3 (T-cells), CD8α (cytotoxic T-cells, NK cells, DC subset) in sequential sections of paraffin-embedded tumors. The *brown staining* illustrates the immune cell markers and the *blue staining* illustrates the cell nuclei. All images show both granulation tissue (*filled arrowhead*) and tumor cell area (*arrowhead*). *left*: untreated tumors, *right*: tumor 3 days after administration of radioimmunoconjugate. Note that the decrease of CD8α-positive cells during this interval is less than that of CD2 and CD3. Scale bars: 100 μm

All T-cell markers decreased in tumors treated with RIT compared with untreated tumors in all areas evaluated, although this was not statistically significant in all cases, see Table 2 and Fig. 1. The potential confounding effect of tumor volume (day 0 or at sacrifice) was evaluated using multiple linear regression, and the estimated time trends were essentially the same as without adjustment for tumor volume (data not shown). The T-cell markers were not expressed in necrotic areas, thus necrotic areas were not included in the following analysis.

**Table 2:** Statistical evaluation of the change in immune cell marker positive cells after administration of RIT. The number of antigen-positive cells at day 0 and the average change in positive cells within tumors treated with RIT (% per day, confidence interval and p value) at different localizations

Antigen	Localization	Positive cells on day 0 median (range)	Average change after admin. of radioimmunoconjugate (95 % confidence interval)	*p* value
CD2	Tumor cell areas	20 (0–80)	−30 % per day (−48 to −5.5)	0.022
	Granulation tissue between tumor cell areas	240 (110–450)	−33 % per day (−51 to −8.2)	0.015
	Granulation tissue surrounding tumor cell areas	370 (110–370)	−36 % per day (−52 to −15)	0.003
CD3	Tumor cell areas	50 (0–150)	−36 % per day (−49 to −18)	0.001
	Granulation tissue between tumor cell areas	350 (150–570)	−24 % per day (−30 to +17)	<0.001
	Granulation tissue surrounding tumor cell areas	480 (130–910)	−29 % per day (−36 to −20)	<0.001
CD8α	Tumor cell areas	90 (30–180)	−10 % per day (−29 to +13)	0.35
	Granulation tissue between tumor cell areas	260 (80–490)	−0.1 % per day (−4.4 to +4.9)	0.95
	Granulation tissue surrounding tumor cell areas	340 (180–530)	−4.7 % per day (−8.8 to +0.41)	0.033
CD68	Tumor cell areas	310 (130–640)	−1.3 per day (−11 to +9.7)	0.80
	Granulation tissue between tumor cell areas	510 (310–710)	+1.1 % per day (−2.8 to +5.3)	0.56
	Granulation tissue surrounding tumor cell areas	560 (200–910)	−9.7 % per day (−14 to −5.1)	<0.001
CD163	Tumor cell areas	160 (60–380)	−13 % per day (−26 to +0.47)	0.057
	Granulation tissue between tumor cell areas	190 (110–310)	+1.1 % per day (−2.8 to +5.3)	0.56
	Granulation tissue surrounding tumor cell areas	200 (100–380)	−4.2 % per day (−9.7 to +1.5)	0.14

**Fig. 2:**
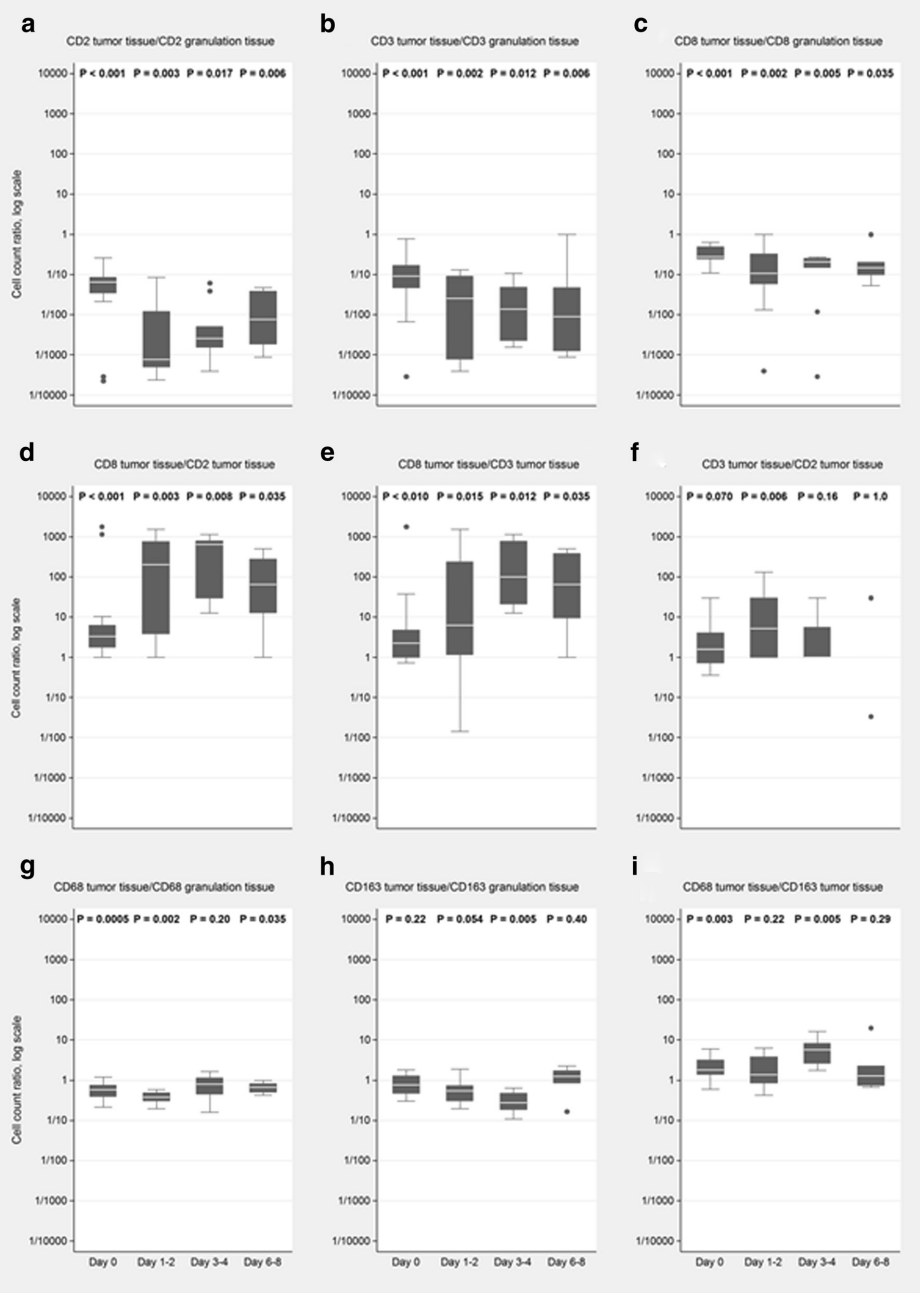
Antigen expression over time. Number of positive cells for the immune cell markers expressed as ratios of expression in tumor cell areas/granulation area between the tumor cell areas (granulation tissue) over time (box plots, stratified by time interval) in tumors treated with RIT (**a**–**c**; **g**–**h**). Number of positive cells for various immune cell marker ratios within the tumor cell areas over time in tumors treated with RIT (**d**–**f**; **i**). A ratio of 1.0 indicates no difference. No box is shown for day 6–8 in F as only two data points were available

All T-cell markers had more positive cells in the granulation tissue (both surrounding and between tumor cell areas) than in the vital tumor cell areas (Fig. 2a–c and Table 2). There were a higher number of positive cells for CD8α than CD2 and CD3 and a tendency towards more positive cells for CD2 than CD3 both in untreated tumors and treated tumors.

### Macrophage markers—CD68 and CD163

**Fig. 3:**
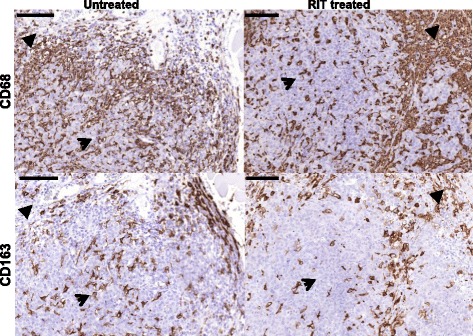
Images of immunohistochemical sections after staining of macrophage markers. CD68 (macrophages, neutrophils, DC, myeloid progenitors, e.g., myeloid suppressor cell) and CD163 (M2, macrophages of pro-tumor type [26, 27]) in sequential sections of paraffin-embedded tumors. The *brown staining* illustrates the immune cell markers and the *blue staining* illustrates the cell nuclei. All images show both granulation tissue (*filled arrowhead*) and tumor cell area (*arrowhead*). *left*: untreated tumors, *right*: tumor 3 days after administration of radioimmunoconjugate. Note the higher number of positive cells for CD68 during this interval than that of CD163. Scale bars: 100 μm

The number of positive cells for both macrophage markers decreased less than T-cell markers in tumors treated with RIT, compared with untreated tumors in all the areas evaluated, see Table 2 and Fig. 3. The potential confounding effect of tumor volume (day 0 or at sacrifice) was evaluated using multiple linear regression, and the estimated time trends were essentially the same as without adjustment for tumor volume (data not shown). The macrophage markers were only expressed as fragments and had no vital cell association in necrotic areas and necrotic areas were not included in the following analysis.

Both macrophage markers tended to have more positive cells in the granulation tissue (both surrounding and between vital tumor cell areas) than in the vital tumor cell areas (Fig. 2g–h and Table 2). There were a higher number of positive cells for CD68 than CD163 both in untreated tumors and treated tumors.

## Discussion

We have demonstrated that the model used in this study has a higher number of positive cells of immune cell markers augmenting immune rejection of tumor cells than immune tolerance of tumor cells,* i.e.* a higher number of positive cells for CD8α than CD2 and CD3 and a higher number of positive cells for CD68 than CD163. The infiltrating immune cells seem to be present to a higher extent in untreated tumors than in tumors treated with RIT. The histological features of untreated tumors and tumors from animals treated with RIT have been described previously, showing that the fraction of granulation tissue increased in tumors treated with RIT (maximum at 4 days p.i.), and that the granulation tissue matured into fibrous tissue which increased throughout the study period [28].

All the immune cell markers were found to have a higher number of positive cells in the granulation tissue than within the vital tumor cell areas. CD8α (antitumor immune cells) decreased less in tumors treated with RIT than the other T-cell markers (CD2 and CD3). The macrophage markers evaluated (CD68 and CD163) decreased less in tumors treated with RIT than the T-cell markers. There was a trend towards a higher number of positive cells for CD68 than CD163 (pro-tumor macrophages) in tumors both before and after the administration of ^177^Lu-BR96.

The observation that the immune cell marker positive cells tended to decrease within the tumors treated with RIT could be explained by the continuous decay of ^177^Lu in the tumor. Lymphocytes are radiosensitive to low doses of radiation [29–31], while macrophages, natural killer (NK) cells, and dendritic cells (DC) are more resistant to irradiation [29, 30]. This could explain why the decrease in CD3 (T-cells) and CD2 (T-cells, B-cells, NK cells) was more pronounced than that of CD8α (cytotoxic T-cells, NK cells, subset of DC), CD68 (macrophages, neutrophils, DC, myeloid progenitors), and CD163 (pro-tumor macrophages, M2).

One advantage of immunohistochemical staining is that it provides information on the localization of the antigens within the structures of the tumor. Others have shown that the localization of immune cell markers is an important predictor of clinical outcome [7, 8, 11, 12, 26, 32–34]. Deschoolmeester et al. showed that a high infiltrating CD3- and CD8-positive cells in cancer cell nests was correlated to improved overall survival in colorectal cancer [8]. Pagès et al. suggested an immunological score based on the quantification of CD3, CD45RO, and CD8 in the core of the tumor and in the invasive margin within the tumor samples. This score was found to be a better predictor of patient survival than the histopathological methods currently used to stage colorectal cancer [11, 12, 33].

The results of immunohistochemical examinations cannot be related directly to cell type, mainly due to the expression of immune cell markers on more than one cell type. The immune cell markers used in the present study and their expression on various immune cells are summarized in Table 1. In the present study, T-cells stained positive for both anti-CD3 and anti-CD2, but CD2 is also expressed by NK and B-cells. CD8α is expressed by cytotoxic T-cells (which also express CD2 and CD3) and NK cells, and a subset of DC. The CD68 antigen is expressed by all macrophages, neutrophils, basophils, DC, and myeloid progenitor cells (e.g., myeloid-derived suppressor cell), while CD163 is expressed by M2 (pro-tumor macrophages) [26, 27].

The therapeutic effect of external irradiation on distant non-irradiated tumor cells (the abscopal effect) has been shown to be at least partly due to the induction of the immune response [29, 31, 35–41]. However, both the radiation dose and the delivery schedule seem to be important for the induction of the abscopal effect [31, 35, 37, 38, 40–43]. It has been demonstrated that these factors can affect the infiltration of CD8α-positive T-cells in tumors [29, 35, 38, 40, 43] and affect the type of the macrophage response (pro-tumor, M2, or anti-tumor, M1) [35, 40, 42, 44]. In a recent study, we demonstrated that early depletion of CD8-positive cells in our rat model treated with RIT seemed not to affect the rejection of the inoculated tumor but increased the number of animals developing metastases [45]. This finding provides evidence that the presence of CD8-positive cells is important in preventing or delaying the development of metastases in this model.

It is of therapeutic interest to evaluate the mechanisms activating the immune system, and to the best of our knowledge this is the first study to evaluate the infiltration of immune cells in tumors after the administration of a radioimmunoconjugate. In future studies, we intend to investigate the effects of combining RIT and immunotherapy in our immunocompetent syngeneic rat tumor model.

## Conclusions

Analysis of the number and localization of immune cell markers within a local tumor at the time of treatment and during rejection after administration of ^177^Lu-BR96 antibodies revealed that all the immune cell markers had a higher number of positive cells in adjacent granulation tissue than in tumor cell areas, and that positive cells for T-cell markers decreased more than positive cells for macrophage markers in tumors treated with RIT. We have thus demonstrated that RIT in this model induce both a shift in the balance to a higher number of cells expressing immune cell markers related to immune rejection than markers related to immune tolerance of tumor cells and also a decrease in cells expressing immune cell markers during RIT compared with untreated tumors.

## Abbreviations

BN: Brown Norway
CR: complete response
DC: dendritic cell
DOTA: 1,4,7,10-tetraazacyclododecane-1,4,7,10-tetraacetic acid
EDTA: ethylenediaminetetraacetic acid
HPLC: high-performance liquid chromatography
HSA: Human serum albumin
ITLC: instant thin-layer chromatography
NK cells: natural killer cells
PBS: phosphate-buffered saline
PI: post-injection
RIT: radioimmunotherapy
